# Flagellate bacteria‐mediated tumour antigen delivery: A novel approach to enhance dendritic cell activation for in situ cancer vaccination

**DOI:** 10.1111/1751-7915.70028

**Published:** 2024-10-18

**Authors:** Wen Xia, Jinhui Wu

**Affiliations:** ^1^ State Key Laboratory of Pharmaceutical Biotechnology Medical School of Nanjing University Nanjing China; ^2^ Chemistry and Biomedicine Innovation Centre Nanjing University Nanjing China; ^3^ Jiangsu Key Laboratory of Molecular Medicine Nanjing University Nanjing China

## Abstract

In situ vaccination is a therapeutic approach aimed at exploiting tumour antigens available at a tumour site to induce tumour‐specific adaptive immune responses. Antigens released from dying tumour cells are assumed to be taken up by activated dendritic cells and presented to T cells that seek out and destroy tumour cells. This process is significantly impeded in the immunosuppressive microenvironment of tumours. There is a growing trend in in situ vaccine strategies that utilize bacteria as natural adjuvants or as factories for cytokines, aiming to enhance the presentation of in situ antigens by antigen‐presenting cells. Recently, a novel approach using flagellate bacteria‐mediated antigen delivery to activate dendritic cells has been proposed. This method actively facilitates the delivery of intratumoral antigens, improving their presentation for in situ cancer vaccination. Here, we highlight how flagellate bacteria‐mediated antigen delivery enhances the immune activation capabilities of in situ vaccines. Meanwhile, we provide perspectives and outlooks on these promising antigen delivery technologies.

## Graphical abstract

Multiple approach enhancing dendritic cell activation via flagellate bacteria‐mediated antigen delivery systems.

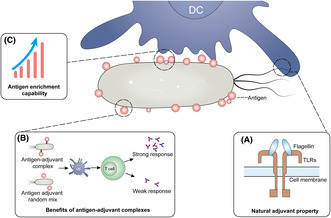

## INTRODUCTION

**FIGURE 1:**
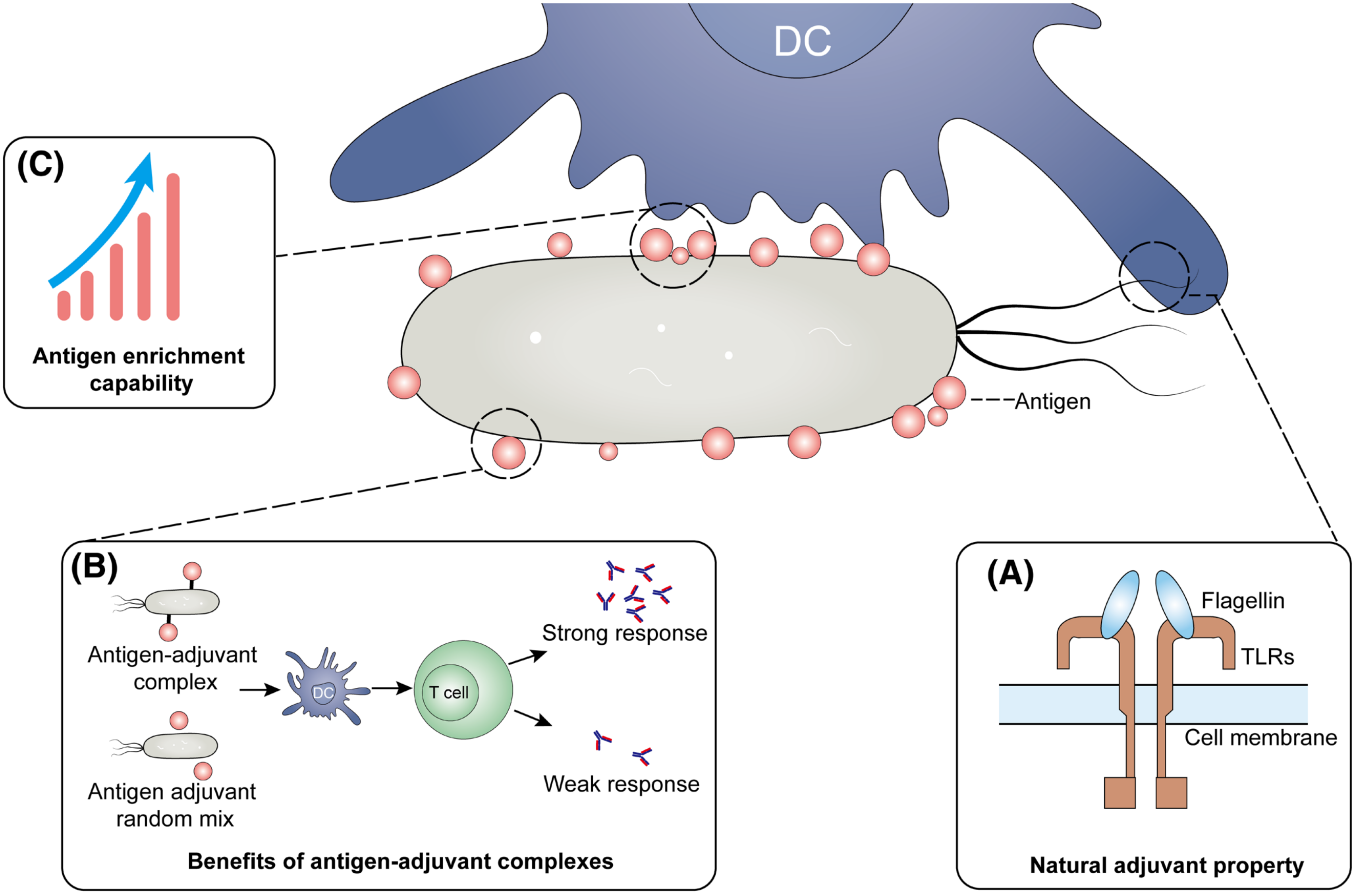
Multiple approaches enhancing dendritic cell activation via flagellate bacteria‐mediated antigen delivery systems. (A) Bacterial flagellin is recognized by Toll‐like receptors, exerting a natural adjuvant effect to activate dendritic cell. (B) Compared to the random mixing of antigens and adjuvants, their conjugation complexes are more advantageous in enhancing the immunogenicity of the antigen and promoting the immune response. (C) Antigens captured by flagellated bacteria increases the local concentration of antigens during dendritic cell uptake.

William Coley conducted the initial administration of heat‐killed bacterial mixtures into cancer patients, noting occasional regression of solid tumours in the 19th century (McCarthy, 2006). Despite the discontinuation of Coley's toxins in medical practice, the concept of introducing bacteria into tumours to elicit an immune response continues to hold significant potential. With the advancement of synthetic biology, leveraging the easily editable nature of bacterial genomes, researchers have engineered bacteria as smarter delivery vehicles for immunotherapeutic payloads. These immunotherapeutic payloads encompass various cytokines such as IL‐2, GM‐CSF (Al‐Ramadi et al., 2009; Sorenson et al., 2008; Yuhua et al., 2001; Zhang et al., 2014), as well as therapeutic antibodies including CD47/PDL1/CTLA4 nanobodies (Gurbatri et al., 2020; Wu et al., 2022). Our group has extended the range of effective payloads to include tumour in situ antigens that can activate adaptive immune responses, taking advantage of the motility of flagellated bacteria to get tumour antigens transported out of irradiated tumours, to functional dendritic cells (DCs) at the tumour periphery and to enhance their activation (Wang et al., 2022). Compared to the traditional strategies involving the intratumoral injection of cytokines to induce DC activation or tumour infiltration, the antigen transfer strategy presents a novel paradigm for activating DCs. This approach effectively circumvents the challenging immunosuppressive microenvironment within the tumour. Previous studies provide detailed evidence of bacterial motility contributing to antigen delivery. Here, we propose that flagellate bacteria, as complex biological components, offer multiple approaches to enhance dendritic cell activation for in situ cancer vaccination. In addition to motility‐mediated antigen transport, their natural adjuvant properties, antigen enrichment capabilities, and the benefits of forming antigen‐adjuvant complexes all contribute to engineered bacteria enhancing the immune activation efficacy of in situ vaccines. Here, we proposed that, as complex biological components, flagellate bacteria offer multiple approaches to enhance dendritic cell activation for in situ cancer vaccination. In addition to flagellate bacteria‐mediated antigen transport, their natural adjuvant property, the benefits of forming antigen‐adjuvant complexes and antigen enrichment capability all contribute to engineered flagellate bacteria enhancing the immune activation efficacy of in situ vaccines (Figure 1).

## NATURAL ADJUVANT PROPERTY

Janeway and Medzhitov first discovered that innate immune cells can detect pathogens through pattern recognition receptors (PRRs), which enhanced our understanding of the innate immune system's role in responding to infections and driving adaptive immune responses (Janeway & Medzhitov, 2002). Bacterial components such as flagellin, lipopolysaccharide (LPS), and bacterial nucleic acids can act as PRR ligands. Upon recognition by PRRs, these components activate innate immune cells, including DCs, macrophages, neutrophils, and natural killer cells, thereby initiating an innate immune response and generating long‐lasting adaptive immunity, known as the adjuvant effect. Four major families of PRRs have been reported: Toll‐like receptors (TLRs), C‐type lectin receptors (CLRs), RIG‐I‐like receptors (RLRs), and nucleotide‐binding oligomerization domain (NOD)‐like receptors (NLRs). Among these, TLRs are the primary receptors recognizing bacterial adjuvant components.

In response to different environments, bacteria have evolved three distinct modes of movement, including swimming, swarming, and gliding motility. The flagellum, as the primary organ of movement, drives bacteria to move through swimming and swarming in fluids and on surfaces. The bacterial flagellum is composed of three main structures: the basal body, the hook and the propeller. The rotation of the flagellar propeller provides direct propulsion for bacterial motility. This propeller is a long, helical filament made up of up to 20,000 subunits of a single protein called flagellin (FliC or FljB in Salmonella species) (Chevance & Hughes, 2008; Wadhwa & Berg, 2022). However, aside from its role in forming the propulsion unit, flagellin has shown tremendous potency as an adjuvant, either in the context of a fusion protein or by co‐administration with antigens (Liu et al., 2011; Skountzou et al., 2010; Taylor et al., 2011). Increasing evidence indicates a collaborative interaction between innate and adaptive lymphocytes. In this context, DCs act as crucial bridges by recognizing and processing antigens, providing activation signals to T cells. Toll‐like receptor 5 (TLR5) is a receptor on DCs that recognizes flagellin. When the extracellular domain of TLR5 binds to flagellin, it gets activated, triggering signalling cascades that enhance the phagocytic and antigen‐presenting abilities of dendritic cells (Amiel et al., 2009; Hayashi et al., 2001; Means et al., 2003; Tsujimoto et al., 2005; West et al., 2004). In particular, TLR5 detects flagellin and activates the MAPK pathway, which activates proteins associated with cytoskeletal remodelling and phagosome formation. These genes include RAC1, F‐actin, Rab5, among others. RAC1, upon activation, functions as a small GTPase that stimulates actin polymerization, thereby bolstering the phagocytic capacity of DCs (Amiel et al., 2009). The MAPK signalling pathway intricately governs the dynamics of F‐actin polymerization and depolymerization. Binding of flagellin protein to TLR5 also promotes F‐actin polymerization, facilitating the formation and maturation of phagosomes (May & Machesky, 2001). Additionally, the MAPK pathway modulates Rab5 activity to promote fusion between phagosomes and lysosomes, thereby augmenting the maturation process of phagosomes (Bucci et al., 1992). In addition, flagellin protein can induce the expression of pro‐inflammatory cytokines such as IL‐1β and TNF‐α. These cytokines, through autocrine and paracrine actions, regulate cytoskeletal dynamics and phagosome formation, thereby enhancing the phagocytic function of DCs (Dinarello, 1996; Locksley et al., 2001).

After engulfing antigens, DCs present processed antigenic peptides to T cells in the form of MHC–peptide complexes. Moldawer and colleagues found that stimulation with flagellin protein upregulates the expression of MHC II on the surface of dendritic cells (DCs) (Tsujimoto et al., 2005), increasing the formation of antigen peptide–MHC complexes on the cell surface. Moreover, flagellin protein regulates the expression of co‐stimulatory molecules such as CD80 and CD86, providing co‐stimulatory signals to T cells that facilitate T cell activation. This enhances the interaction between DCs and T cells, thereby further improving the efficiency of antigen presentation (Mizel & Bates, 2010). Overall, bacteria‐derived flagellin, as natural adjuvant, can enhance the antigen‐presenting capability of dendritic cells (DCs) and promote T cell activation.

## THE BENEFITS OF FORMING ANTIGEN‐ADJUVANT COMPLEXES

Flagellated bacteria form antigen‐adjuvant complexes after capturing antigens, creating an antigen and adjuvant co‐delivery system. Compared to the simple mixing of antigens and adjuvants, their conjugation or co‐delivery via nanoparticles is more advantageous in enhancing the immunogenicity of the antigen and promoting the immune response (Wang & Xu, 2020). This may be because the complex form facilitates the simultaneous action of antigen and adjuvant on the same DC. Using thiol chemistry (SPDP method), ovalbumin (OVA) antigen was covalently linked to an adjuvant N‐trimethyl chitosan (TMC) to form a complex. The uptake of the resulting TMC‐OVA complex by dendritic cells (DCs) and its effect on DC maturation were evaluated in vitro. Findings reveal that DC uptake of TMC‐OVA conjugates was more than five times greater compared to a simple mixture of OVA and TMC. In vivo experiments further demonstrated that mice immunized with the TMC‐OVA conjugate generated OVA‐specific IgG titers that were 1000 times higher than those observed in mice immunized with either OVA alone or a physical mixture of TMC and OVA (Slütter et al., 2010). Ag85B and HspX are two significant virulence protein antigens of Mycobacterium tuberculosis. When these antigens are covalently conjugated with Arabinogalactan (AG)‐poly(I:C) to form a complex and used to immunize mice, they produce high titers of Ag85B and HspX‐specific antibodies. Additionally, this complex enhances T cell proliferation and the secretion of cytokines IFN‐γ, TNF‐α, IL‐2, IL‐4, and IL‐10. Notably, the immune activation effect of the complex form of the vaccine is approximately four times greater than that of a simple mixture of Ag85B and HspX antigens with an adjuvant (Huang et al., 2016).

Non‐protein adjuvants are typically covalently linked to antigens to form complexes, whereas protein adjuvants such as bacterial flagellin and cytokines are predominantly delivered together with antigens through genetic fusion. For example, TLR5 ligand flagellin achieves codelivery with antigen by gene fusion. The C‐terminus of flagellin from salmonella typhimurium was fused with the HA1 fragment of H1N1 influenza virus and produced in E. coli strain BL21 (DE3) to obtain the adjuvant–antigen codelivery vaccine VAX125 (Taylor et al., 2011; Treanor et al., 2010). By changing the coupling site between flagellin and HA, the fusion protein vaccine VAX128B (flagellin N‐terminal fused with HA1) and VAX128C (flagellin N‐terminus and C‐terminus fused with HA1) were obtained, which showed the same immunogenicity but greater safety. All three of the VAX128 vaccine constructs were immunogenic in the rabbit model with over 4‐fold increases in HA1 antibody titre observed 7 days post the booster immunization at all dose levels (Taylor et al., 2012). A phase I clinical trial demonstrated that a small dose (1 μg) of flagellin‐antigen complex has good immunogenicity and safety.

Heat shock proteins (HSPs) are typically produced in response to stress and possess certain immunomodulatory properties, making them useful as vaccine adjuvants. A codelivery system that fuses HSP‐70 with HIV‐1 p24 proteins has been shown to induce stronger cellular immunity compared to a simple antigen–adjuvant mixture (Krupka et al., 2015). Additionally, using a gene fusion method, mouse Hsp70 has been fused with the tumour antigen MAGE‐A1 to create an adjuvant–antigen complex vaccine, which significantly elicits humoral immune response and delays tumour growth and compared to those without adjuvant or with a simple adjuvant–antigen mix (Jiang et al., 2013). Similarly, a fusion protein combining human papilloma virus 16 (HPV16) mE6/mE7 with TBHSP‐70 proteins has demonstrated good antitumor effects as a therapeutic vaccine (Qian et al., 2006).

Overall, previous studies have consistently demonstrated the clear advantages of antigen‐adjuvant complexes codelivery systems that integrate antigen and adjuvant into a complex, whether through physical, chemical, or fusion protein methods, prove more effective in enhancing antigen immunogenicity compared to simple mixtures. When antigens and adjuvants are merely mixed, they tend to separate, diminishing their collective impact on immune cells and thereby weakening the immune response. In the flagellate bacteria‐mediated tumour antigen delivery system, the absorption of antigens leads to the formation of a multivalent adjuvant‐antigen complex. This feature augments dendritic cell activation through bacterial motility, thereby enhancing the efficacy of in situ cancer vaccination.

## ANTIGEN ENRICHMENT CAPABILITY

The initial step in the flagellin‐mediated antigen delivery strategy involves the capture of released antigens within the tumour microenvironment. Simultaneously, these antigens are enriched on the surface of the bacteria. This enrichment increases the local concentration of antigens, thereby enhancing the efficiency of antigen presentation by DCs. Wang et al.'s research supports this concept, as they were the pioneers in proposing the concept of antigen capture and developed a series of nanoparticles with antigen‐adsorbing capabilities (Wang et al., 2022). These nanoparticles have been demonstrated to enrich hundreds of antigens, thereby enhancing antigen presentation by DCs and driving a robust adaptive immune response (Min et al., 2017). As natural nanoparticles secreted by gram‐negative bacteria, outer membrane vesicles (OMVs) have garnered significant attention as potential platforms for vaccine delivery (Chen et al., 2021). Maleimide modified OMVs have also been reported to possess the ability to enrich a range of tumour antigens. These enriched antigens are delivered to dendritic cells (DCs), enhancing tumour antigen presentation. This strategy amplifies the immune activation effect triggered by photodynamic therapy (Li et al., 2022). The bacterial surface layer protein (SLP) can be attached to cationic liposomes through an electrostatic interaction with the carboxyl groups of the SLP. This method enables cationic liposomes to enrich bacterial antigens, thereby promoting the production of specific antibodies (Zhou et al., 2023). In conclusion, the encouraging results of the nanoscale carrier antigen enrichment effect in immune activation support our understanding of its positive contribution in the flagellate bacteria‐mediated tumour antigen delivery system.

## PERSPECTIVE AND OUTLOOK

In situ cancer vaccination has made a significant impact on clinical cancer treatment (Saxena et al., 2021). In contrast to traditional neoantigen vaccines, the in situ vaccine approach generates the vaccine directly within the tumour microenvironment (TME) using antigens from dead or dying tumour cells. This method eliminates the need for antigen identification and isolation, thereby reducing delays and costs associated with producing personalized vaccines externally (Fucikova et al., 2020; Shae et al., 2020). As a result, it provides an existing strategy for cancer vaccine manufacture. Furthermore, tumour cells easily evade immune responses mediated by traditional monoclonal antigen vaccines. In contrast, in situ vaccines are essentially multivalent vaccines capable of eliciting polyclonal immune responses against antigens expressed by different cancer cell subclones, offering a potential approach to address tumour heterogeneity (Dagogo‐Jack & Shaw, 2018). An essential requirement for the effective implementation of in situ vaccines is the efficient processing and presentation of antigens by dendritic cells. However, this critical process is often impeded within the immunosuppressive tumour microenvironment, thereby limiting the wider application of in situ cancer vaccines. Consequently, there is an urgent demand for new delivery technologies capable of enhancing dendritic cell activation and the processing of tumour antigens. Responding to this pressing need, a novel approach leveraging flagellate bacteria to enhance dendritic cell activation for in situ cancer vaccination has been proposed. While this approach has shown some promising results, several considerations still require careful evaluation and resolution.

Upon stimulation by cell death inducers such as ionizing radiation and chemotherapeutic drugs, tumour cells release antigens into the tumour microenvironment (TME). Along with tumour antigens, self‐antigens are also released from the dying cells (Li et al., 2022; Wang et al., 2022). The currently proposed flagellate bacteria antigen delivery vectors or bacteria‐derived nanocarriers capture antigens with a non‐selective manner, delivering both tumour and self‐antigens to antigen‐presenting cells (APCs). However, the processing of self‐antigens by APCs can induce immune tolerance, which is undesirable for effective in situ vaccination in which the aim is to generate high‐intensity and specific antitumour immune responses (Makkouk & Weiner, 2015). Moreover, the non‐specific capture of antigens poses an additional challenge: competitive occupation of binding sites on the vectors limits the enrichment of genuine tumour antigens. To overcome these hurdles, there is an urgent need to develop flagellate bacteria delivery technologies capable of selectively capturing tumour antigens and delivering them to APCs. This approach aims to induce potent tumour antigen‐specific immune responses, enhancing the efficacy of in situ vaccination strategies.

In addition to serving as antigen delivery vectors, many bacteria‐based tumour treatments have been proposed. Before clinical use, these methods must address common safety concerns. Although attenuated strains of *Clostridium* and *Salmonella* have been proven to be non‐pathogenic in various animal species (Low et al., 1999; Thamm et al., 2005) and human trials (Heimann & Rosenberg, 2003; Nemunaitis et al., 2003; Toso et al., 2002), any retained virulence factors could still be pathogenic to immunocompromised late‐stage cancer patients. Hundreds of chemotaxis receptors found in bacteria sense environmental compounds and mediate their directional movement. Due to the presence of tumour‐specific secreted metabolites and proteins, this chemotaxis machinery also enables bacteria to colonize tumours (Kasinskas & Forbes, 2007; Song et al., 2018; Zhang & Forbes, 2015). Maximizing the targeting efficiency of bacteria to tumour sites can reduce the dosage of bacterial drugs injected, while ensuring an effective concentration within the tumour. This can be achieved by enhancing tumour targeting through the genetic modification of endogenous chemoreceptors, selectively controlling bacterial proliferation within tumours, or improving the directed chemotactic movement of bacteria toward tumours (Kasinskas & Forbes, 2007). It is crucial to identify which virulence factors are essential for bacterial targeting and which are responsible for unnecessary toxicity. This can be achieved by screening knockout models of the pathogenicity genes that facilitate immune evasion, promote intracellular replication, and stimulate cytokine synthesis. Combining genetic engineering and synthetic biology techniques to knock out or regulate the expression of these virulence factors can enhance bacterial safety, which has been implemented in the attenuated *Salmonella typhimurium* (VNP20009) (Toso et al., 2002).

In all, an increasing number of delivery technologies are being used to enhance in situ vaccines. Flagellate bacteria‐mediated antigen delivery strategies have emerged due to their encouraging effects on dendritic cell activation and antigen presentation. Flagella‐mediated motility enables bacteria to transfer antigens while their adjuvant effect further activates dendritic cells. Engineering modifications to the bacteria confer properties for antigen enrichment and adjuvant‐antigen complex assembly. This “four‐in‐one” mechanism maximizes the activation of dendritic cells and the immunogenicity of the antigens. With continuous improvements in the safety and specificity of antigen capture, flagellate bacteria‐mediated tumour antigen delivery systems hold promise for broader application in both basic immunology research and clinical settings for in situ vaccination.

## AUTHOR CONTRIBUTIONS

**Wen Xia:** Writing – original draft; software; conceptualization; writing – review and editing; visualization. **Jinhui Wu:** Conceptualization; writing – review and editing.

## CONFLICT OF INTEREST STATEMENT

The authors declare no conflicts of interest.

## Data Availability

The data that support the findings of this study are available from the corresponding author upon reasonable request.
